# Protein profiling of alpha-fetoprotein producing gastric adenocarcinoma

**DOI:** 10.18632/oncotarget.8571

**Published:** 2016-04-04

**Authors:** Liang He, Fei Ye, Linlin Qu, Daguang Wang, Miao Cui, Chengguo Wei, Yanpeng Xing, Peng Lee, Jian Suo, David Y. Zhang

**Affiliations:** ^1^ Department of Gastrointestinal Surgery, First Hospital of Jilin University, Changchun, China; ^2^ Department of Pathology, Icahn School of Medicine at Mount Sinai, New York, NY, USA; ^3^ Department of Clinical Laboratory, First Hospital of Jilin University, Changchun, China; ^4^ Department of Nephrology, Icahn School of Medicine at Mount Sinai, New York, NY, USA; ^5^ Department of Pathology, New York University School of Medicine, New York, NY, USA

**Keywords:** AFP, gastric cancer, signaling pathways, survival analysis

## Abstract

Alpha-fetoprotein (AFP) producing gastric adenocarcinoma is considered as a rare subtype of gastric adenocarcinoma. Compared with AFP non-producing gastric adenocarcinoma, our study and other previous studies showed that AFP producing gastric adenocarcinoma is more aggressive and prone to liver metastasis. Using the Protein Pathway Array, 11 of out of 286 proteins tested were found to be differentially expressed between AFP producing (n=32) and AFP non-producing (n=45) gastric adenocarcinoma tissues. In addition, the high level expression of XIAP and IGF-Irβ in gastric adenocarcinoma tissues was independent factors for poor prognosis in AFP producing gastric adenocarcinoma patients. A risk model based on the XIAP and IGF-Irβ expression levels can separate AFP producing gastric adenocarcinoma patients into 2 subgroups and each subgroup had a distinct set of signaling pathways involved. In conclusion, AFP producing gastric adenocarcinoma is a heterogeneous cancer with different clinical outcomes, biological behaviors and underlying molecular alterations.

## INTRODUCTION

Alpha-fetoprotein (AFP), the equivalent of an albumin in serum in adults, is produced in the fetal liver and yolk sac [1, 2]. Elevated serum AFP level in adults is considered as abnormal and serum AFP level is frequently used as a suitable biomarker for diagnosing and monitoring hepatocellular carcinoma (HCC) and yolk sac tumor. In addition, a few other tumors including gastric adenocarcinoma can also produce AFP.

Since initial report by Bourreille et al in 1970 [3], AFP producing gastric adenocarcinoma attracted significant attention and more cases were subsequently reported [4]. Currently, AFP producing gastric adenocarcinoma is considered as a rare subtype of gastric adenocarcinoma. Compared with AFP non-producing gastric adenocarcinoma, previous studies [5–8], including our study [9], showed that AFP producing gastric adenocarcinoma is more aggressive with high incidence of liver metastasis and poor prognosis.

In the past years, the biological behavior and molecular characteristic of AFP-producing gastric adenocarcinoma has been studied. The previous reports showed that Ki-67, c-Met, vascular endothelial growth factor (VEGF), STAT3, hepatocyte growth factor and its receptor were highly expressed in AFP producing gastric adenocarcinoma tissues and cell lines as compared with AFP-negative gastric adenocarcinoma [10–14]. Those factors play important roles in cell mitosis, proliferative activity, migration and tumor progression and they may potentially serve as molecular markers of AFP producing gastric adenocarcinoma. However, how these factors and their associated signaling pathways regulate the aggressive behavior of AFP producing gastric adenocarcinoma remain to be fully established. Furthermore, since AFP-producing gastric adenocarcinoma is not homogeneous in terms of clinical outcome and molecular characteristics, it is critical to identify biomarkers that can further subcategorize AFP-producing gastric adenocarcinoma.

In the current study, we investigated the expression of 286 functionally important proteins and phosphoproteins in AFP producing and AFP non-producing gastric adenocarcinoma using high-throughput protein pathway array (PPA) [15]. Eleven differentially-expressed proteins were identified and several of them correlate with relapse-free survival (RFS) and overall survival (OS) of AFP producing gastric adenocarcinoma.

## RESULTS

### Preoperative serum AFP levels between AFP producing and non-producing gastric adenocarcinoma patients

The clinicopathological characteristics and follow-up results of the patients were summarized in Table 1. The average serum AFP level was 2.46±1.00 ng/L (range: 0.39 to 5.09, standard error: 0.14) in the AFP non-producing group, and was 117.3 ng/L (range: 7.19 to 1210, standard error: 44.99) in the AFP producing group. Significant difference was observed in serum AFP level between the two groups (p=0.03). However, no significant differences were observed between the two groups for other tumor markers, including CA-724, CA199, CEA and CA125.

**Table 1:** Clinicopathological characteristics of AFP producing and non-producing gastric cancer

	AFP producing GC	AFP non-producing GC	p Value
**No. of patients**	32 (41.6%)	45 (58.4%)	N/A
**Serum AFP (ng/ml)**	117.3±254.5	2.5±1.0	0.030*
**Mean age (years)**	60.2±11.1	63.1±11.3	0.262
**Gender (M/F)**	26/6	33/12	0.299
**Drink (Y/N)**	9/23	11/34	0.456
**Smoke (Y/N)**	15/17	14/31	0.121
**Tumor size (cm)**	6.2±2.5	5.7±2.1	0.379
**Vascular invasion (Y/N)**	28/4	39/6	0.598
**Tumor site**			
Proximal (cardia or fundus)	12	17	0.586
Distal (antrum or corpus)	20	28	
**Histology** (poor/well or moderate)	23/9	34/11	0.458
**Curative surgery (Y/N)**	27/5	43/2	0.110
**TNM stage**			
Ib	1	1	0.226
IIb	4	6	
IIIa	2	0	
IIIb	5	13	
IIIc	15	23	
IV	5	2	
**Lymphatic invasion (Y/N)**	24/8	40/5	0.100
**Outcome**			
Relapse (Y/N)	27/5	28/17	0.029*
Liver metastasis (Y/N)	20/12	5/40	0.001*
**Status at last following up**			
Deceased	26	25	
Live	6	20	0.016*

* p<0.05

### High level of serum AFP was an independent prognostic factor

The median follow-up was 24.6 months (range, 2–64.4 months). Fifty-five patients had relapsed, among them, 20 with local recurrence and 35 with metastasis at distant sites including 25 with liver metastases. In AFP producing gastric adenocarcinoma group, 27 patients had relapsed [84.4% (27/32)] compared with 28 in AFP non-producing gastric adenocarcinoma group [62.2% (28/45)] (p=0.029).

Mean relapse free survival (RFS) was 9 months (95%CI: 6.6-11.6 months) in the AFP producing gastric adenocarcinoma group, compared with 30 months (95% CI: 6.6-53.4months) in the AFP non-producing gastric adenocarcinoma group (log-rank p=0.030). In AFP producing gastric adenocarcinoma group, 20 patients had liver metastasis [62.5% (20/32)] compared with 5 in AFP non-producing gastric adenocarcinoma group results [11.1% (5/45)] (p=0.001).

**Figure 1:**
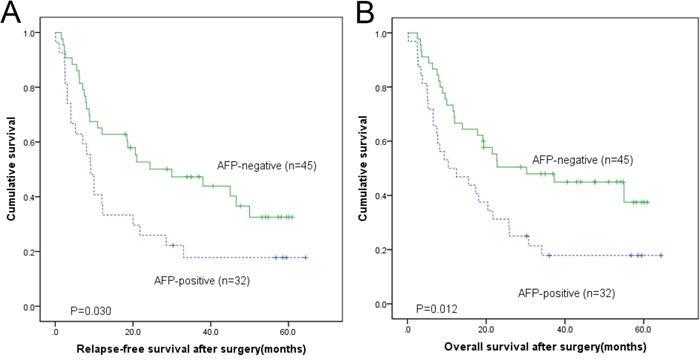
The Kaplan–Meier and log-rank survival analysis showed that the AFP producing gastric adenocarcinoma were associated with a poorer relapse-free **A.** and overall **B.** survival after surgery.

Mean overall survival (OS) was 21.5 months (95% CI, 13.9-29.0 months) in AFP producing gastric adenocarcinoma group, compared with 34.5 months (95% CI, 27.4-41.7 months) in the AFP non-producing gastric adenocarcinoma group. Kaplan-Meier survival analyses showed that AFP non-producing gastric adenocarcinoma group correlated with significant favorable RFS and OS (log-rank p=0.030 and p=0.012, respectively) (Figure 1).

Univariate Cox analyses showed that the following factors were associated with unfavorable RFS, including lymph node metastasis [hazard ratio (HR): 2.754; 95% CI: 1.087-6.974; p=0.033], higher TNM stage (HR: 1.553; 95% CI: 1.153-2.093; p=0.004), and AFP positivity (HR: 1.863; 95% CI: 1.053-3.297; p= 0.033). Furthermore, the following factors were associated with unfavorable OS, including the lymph node metastasis (HR: 2.704; 95% CI: 1.071-6.825; p=0.035), distant metastasis (HR: 5.323; 95% CI: 2.282-12.418; p=0.000), higher TNM stage (HR: 1.996; 95% CI: 1.409-2.827; p=0.000), and AFP positivity (HR: 2.004; 95% CI: 1.154-3.478; p= 0.014) (Supplementary Table 1, 2).

Multivariate Cox analyses indicated that the following factors were associated with unfavorable RFS, including gender (HR: 2.115; 95% CI: 1.031-4.338; p=0.041), higher TNM stage (HR: 1.689; 95% CI: 1.021-2.796; p=0.041), and AFP positivity (HR: 3.006; 95% CI: 1.560-5.791; p=0.033). Furthermore, tumor site (HR: 2.242; 95% CI: 1.052-4.1774; p=0.036), higher TNM stage (HR: 2.441; 95% CI: 1.616-3.688; p=0.000), and AFP positivity (HR: 2.9441; 95% CI: 1.573-5.509; p= 0.001) were associated with unfavorable OS. Finally, the multivariate Cox analyses suggested that serum AFP level and higher TNM stage were independent prognostic factors for RFS and OS.

### Correlation of protein expression and canonical pathways with clinical characteristics of AFP producing gastric adenocarcinoma

To explore the relationship between protein expression and clinical characteristics of AFP producing gastric adenocarcinoma (i.e. gender, age, histological grade, vascular invasion, tumor size, drink and smoking), the unpaired t test and SAM analysis were performed. Table 2 showed the results of differentially expressed proteins correlated with various clinicopathological characteristics with statistical significance (p<0.05 or q<0.05). For example, ADAM-10 was up-regulated in AFP producing gastric adenocarcinoma tumor tissues with poor histology grade and distal location, while DPYD was down-regulated in the tumor tissues with moderate histology grade and without vascular invasion. No correlations were found between protein expression and drinking, smoking or age.

**Table 2:** Correction between protein expression and clinical characteristics

Clinicopathological characteristics	Differentially expressed proteins*	
	up-regulated	down-regulated
**Gender** (male vs female)	Cdk6, MetAP-2	Bak, Calretinin, Flk-2
**Tumor location** (distal vs proximal)	ADAM-10	Notch4, Caspase-1
**Histology grade** (poor vs moderate)	ADAM-10, FOXM1 IGF-Irβ, RANKL, Cyclin B1	DPYD, Maspin
**Tumor size** (≥5cm vs <5cm)	Vimentin, Endoglin, Autotaxin	NOS2
**Vascular invasion** (present vs absent)	Annexin A1, SK3	DPYD, p-PKC α/βII
**Hepatic metastasis** (present vs absent)	Bcl-2	eIF4B, IL-6, Rho A

* up-regulated indicates an increased protein expression and down-regulate indicates a decreased protein expression in the former group when compared with the latter group in each category.

**Figure 2:**
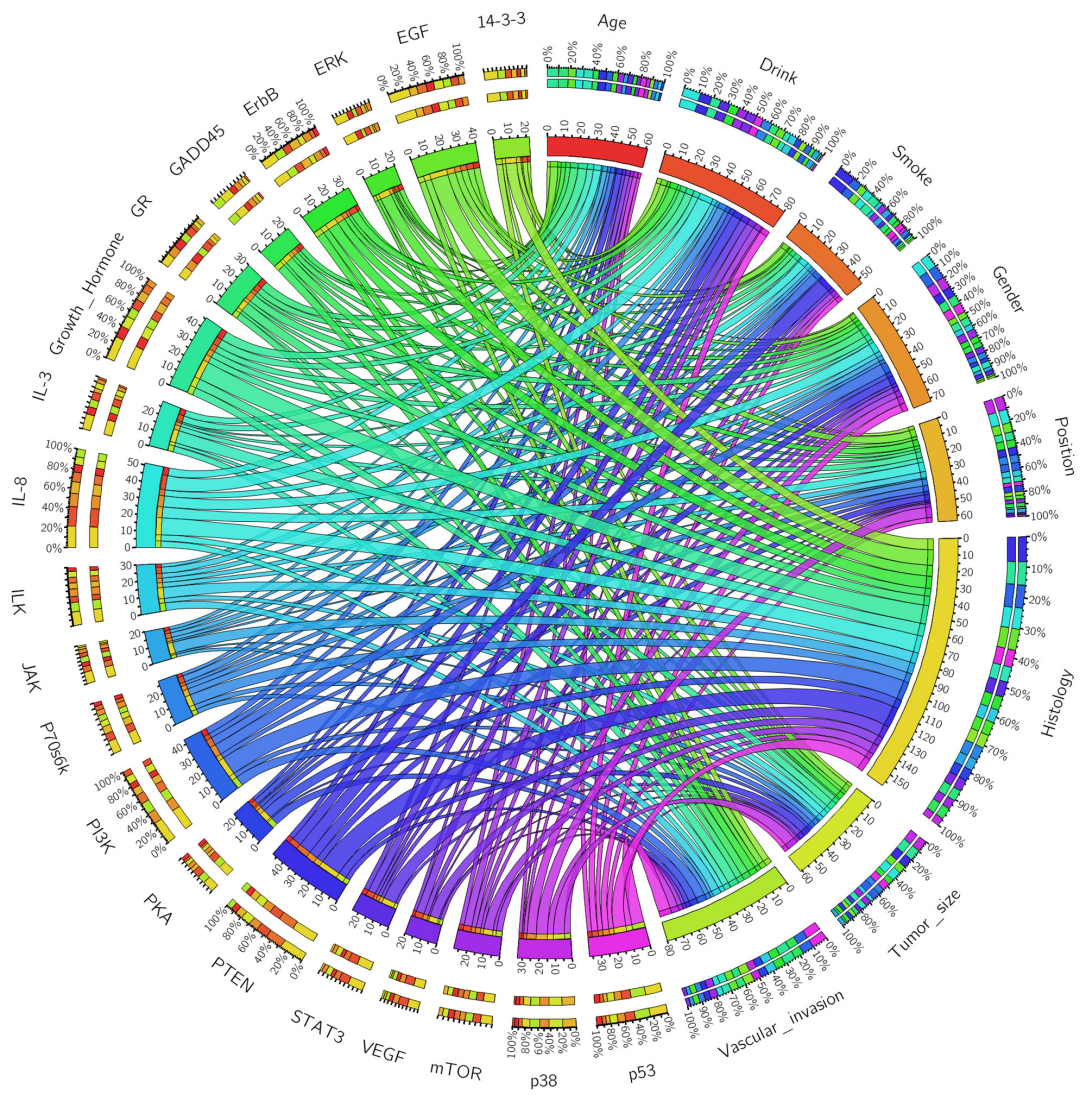
The circos plot depicting the correlation between cellular signaling pathways and the clinicopathological categories in AFP producing gastric adenocarcinoma Each signaling pathway and clinicopathological category are assigned with a specific color. The association between the clinicopathological categories and signaling pathways are depicted by the arcs. The area of each colored ribbon depicts the proportion of the signaling pathway contributes to a particular clinicopathological category.

As previously described in method, data (gene names and fold changes) of the proteins in each clinicopathological category were uploaded into IPA for further functional annotation and pathway analysis. IPA ranked the most relevant canonical signaling pathways associated with each clinicopathological characteristics. The results of the IPA analyses were expressed in a circos plot (Figure 2). Among all 8 clinicopathological categories, the tumor histology occupied the greatest proportion of the distribution, suggesting that it is the most important clinical factor. Among 20 pathways altered in gastric adenocarcinoma, the PI3k, PTEN, IL-8 and growth hormone signaling pathways were affected most, suggested that they play important roles in pathogenesis of AFP producing gastric adenocarcinoma.

### Differentially expressed proteins in AFP producing and non-producing gastric adenocarcinomas

**Figure 3:**
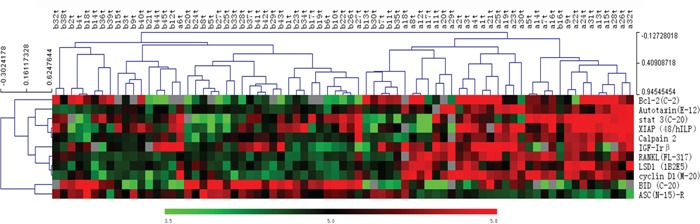
Hierarchical clustering analysis of 11 differentially expressed proteins between 32 AFP producing and 45 AFP non-producing samples The number in each column represents the sample number (a=AFP producing, b=AFP non-producing, t=tumor). Each row represents a protein. The color scale showed the level of expression. Red indicates high expression, green indicates low expression, and gray indicates no expression.

Out of 286 proteins studied, 119 were detected in all 77 tumor samples (32 AFP producing and 45 AFP non-producing), and 11 of 119 proteins were found to be differentially expressed between the two groups based on the unpaired t test and SAM analysis (p<0.05 and q<5%, Supplementary Table 3). Among these 11 proteins, 9 were up-regulated in AFP producing gastric adenocarcinoma, including cyclin D1, RANKL, LSD1, Autotaxin, Calpain2, stat3, XIAP, IGF-Irβ, and Bcl-2. Two proteins were down-regulated in AFP producing gastric adenocarcinoma, including ASC-R and BID. To visualize the expression pattern of the 11 proteins in relation to AFP status, a two-way hierarchical clustering analysis was performed using Multi Experiment Viewer. An obvious difference in protein expression were observed between AFP producing and AFP non-producing gastric adenocarcinomas, although 3 samples were misclassified (Figure 3), suggesting that non-AFP producing gastric adenocarcinoma is a distinct entity.

### Association between protein expression and survival

**Figure 4:**
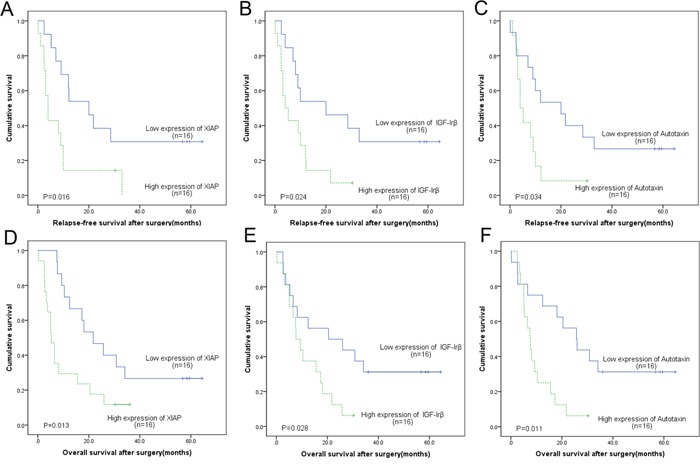
In AFP producing group, the Kaplan–Meier and log-rank survival analysis of the relapse-free survival A-C. and overall survival D-F. showed that the group with high level expression of XIAP A, D., IGF-Irβ B, E. and Autotaxin C, F. was associated with poor prognosis

To determine the association of the differentially expressed proteins with RSF and OF in the two groups, the Kaplan-Meier and log-rank survival analysis was conducted. In AFP producing gastric adenocarcinoma group, poor RSF and OS were associated with high levels of expression of XIAP (log rank, p=0.016 and p=0.013, respectively), IGF-Irβ (p=0.024 and p=0.028) and Autotaxin (p=0.034 and p=0.011) (Figure 4). The other 8 differentially expressed proteins did not show any association with RSF or OS. In contrast, in AFP non-producing gastric adenocarcinoma group, none of 11 differentially expressed proteins, XIAP IGF-Irβ and Autotaxin included (Supplementary Figure 1), were demonstrated association with RSF and OS.

Both univariate and multivariate Cox proportional hazard regression analyses were performed on clinicopathological parameters and these 11 differentially expressed proteins. In univariate analyses of AFP-producing gastric adenocarcinoma group, higher TNM stage and increased expression of XIAP, IGF-Irβ and Autotaxin were found to be correlated with poor RFS and OS (Supplementary Tables 4 and 5). In multivariate analyses, only higher TNM stage and increased expression of XIAP and IGF-Irβ were independent prognostic factors for relapse-free survival and overall survival, including XIAP (RFS: HR, 9.646; 95% CI, 1.824-50.996, pp=0.008; OS: HR, 8.468; 95% CI, 2.246-31.932, p=0.002), IGF-Irβ (RFS: HR, 7.955; 95% CI, 1.390-45.538, p= 0.020; OS: HR, 9.492; 95% CI, 1.630-55.274, p= 0.012), and TNM stage (RFS: HR, 4.194; 95% CI, 1.286-13.686, p=0.017; OS: HR, 2.907; 95% CI, 1.052-8.034, p= 0.040).

In univariate analyses of AFP non-producing gastric adenocarcinoma group, lymph node metastasis, higher TNM stage and increased expression of Bcl-2 were found to be correlated with poor RFS and OS (Supplementary Tables 6 and 7). In multivariate analyses, higher TNM stage (RFS: HR, 2.070; 95% CI, 1.044-4.105; p=0.037; OS: HR, 2.905; 95% CI, 1.153-7.318, p=0.024) and increased expression of BCL-2 (RFS: HR, 0.338; 95% CI, 0.139-0.821, p= 0.017; OS: HR, 0.317; 95% CI, 0.122-0.827, p=0.019) were independent prognostic factors for poor relapse-free survival and overall survival.

### Risk model separated AFP producing gastric adenocarcinoma patients into 2 distinct groups

**Figure 5:**
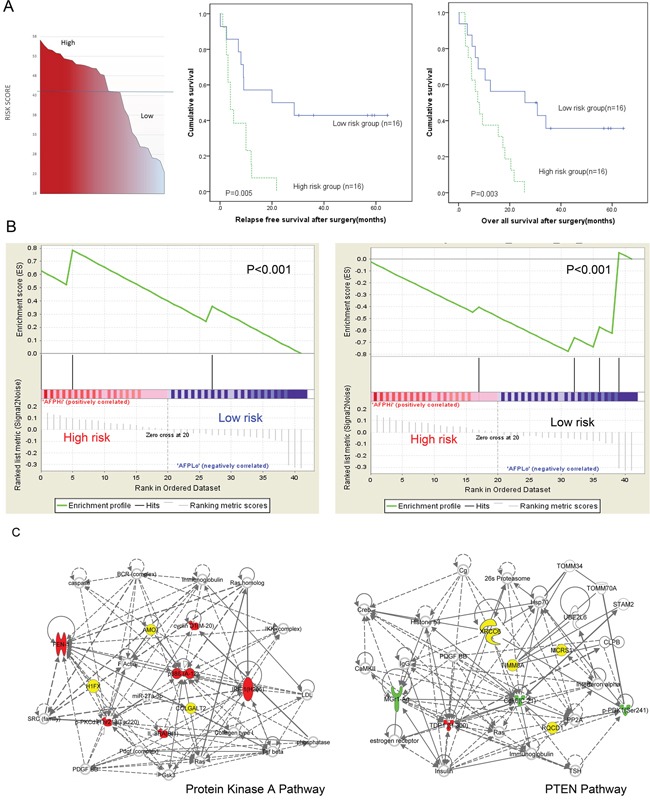
Distinct subgroups of AFP producing gastric adenocarcinomas **A.** The Kaplan–Meier and log-rank survival analysis showed risk score could stratify the AFP producing gastric adenocarcinoma patients into 2 distinct subgroups, with the high risk score group associated with a poorer prognosis both in relapse free(left) and overall survival (right). **B.** High (left) and low risk (right) score groups associated with 2 different enriched gene sets. **C.** The pathways and molecular signatures involved in high and low risk score groups are different: protein kinase A pathway play a key role in high risk score group(left) while PTEN pathway is involved in low risk group(right). Enriched genes and proteins identified by PPA in each network are depicted. (Red: up-regulated proteins; Green: down-regulated proteins; Yellow: enriched genes; Blank: connection genes).

In AFP producing gastric adenocarcinoma group, the risk scores were calculated based on 3 independent factors including XIAP, IGF-Irβ and TNM stage, as well as their corresponding regression coefficients. The risk score for each gastric adenocarcinoma patient was calculated using the formula as described previously: Risk Score=*H_1_* × *X_1_* + *H_2_* × *X_2_* + …+ *H_p_* × *X_p_*, where *X_1_*…*X_p_* were the independent variables and *H_1_…H*_p_ were their hazard ratios, which were determined by multivariate Cox regression analysis. [16, 17]. By using the risk scores, the patients with AFP producing gastric adenocarcinoma was able to classify into high or low risk groups, separated on 50% median of the risk scores. The patients with higher risk scores associated with poorer survival as compared with those with lower risk scores, suggesting 2 distinct subgroups in AFP producing gastric adenocarcinoma (Figure 5A). The clinicopathological data for the patients in each groups were summarized in Supplementary Tables 8.

To further investigate the pathways and molecular signatures associated with each risk group in AFP producing gastric adenocarcinoma, IPA and GSEA were performed. In high risk score group, GSEA gene analysis showed a significant enrichment of a set of genes (p<0.0001), including GLT25D2, AMOT and H1FX. These genes are involved in Protein kinase A (PKA) pathway [−log (p) = 12.60] (Figure 5B and 5C). While in the low risk score group, GSEA gene analysis showed a significant enrichment of a gene set (p<0.0001), including RQCD1, MCRS1, XRCC6 and TTMM8A. This gene set was associated with PTEN pathway [−log (p) = 8.47].

## DISCUSSION

Gastric adenocarcinoma, like other cancers showed significant heterogeneity, clinically, histologically and genetically. AFP producing gastric adenocarcinoma is a rare group gastric adenocarcinoma with frequent liver metastasis and poor prognosis. The prevalence of AFP producing gastric adenocarcinoma has been reported to be 1.3∼6.3%, with high level of serum AFP being an independent prognostic factor [5–9]. To explain the different biological behavior, cellular factors, such as Ki-67, c-Met, vascular endothelial growth factor-C (VEGF-C), STAT3, hepatocyte growth factor and its receptor, have been investigated in AFP producing gastric adenocarcinoma and cell lines [10–14]. However, the exact molecular mechanism of the aggressive behavior is far from clear. In an attempt to correlate protein expression with clinical behaviors and to understand the signaling pathways, we applied Protein Pathway Array technology to identify proteins altered in AFP-producing gastric adenocarcinoma.

Compared with previous studies [10–14], this study investigated much more signaling related proteins simultaneously (a total of 286) in AFP-producing gastric adenocarcinoma. Eleven proteins were found to be differentially expressed in AFP-producing gastric adenocarcinoma in this study and these proteins play important roles in cell signaling pathways. Dysregulation of stat3 and Bcl-2 in AFP-producing gastric adenocarcinoma has been reported in a previous study [14]. However, to our knowledge, dysregulation of cyclin D1, RANKL, LSD1, Autotaxin, Calpain2, XIAP, IGF-Irβ, ASC-R and BID in AFP producing gastric adenocarcinoma has not been reported before. More importantly, our study showed that the high level expression of XIAP and IGF-Irβ were independent prognostic factors and correlated with poor survival in AFP producing gastric adenocarcinoma patients but not in the AFP non-producing patients.

In the IAP family (inhibitor of apoptosis), XIAP (X-linked inhibitor of apoptosis) is the most potent and versatile inhibitor of apoptosis and caspases [18]. Previous studies demonstrated that XIAP is up-regulated in many gastric adenocarcinoma cells [19, 20] and XIAP inhibitors can increase apoptosis and enhance sensitivity of gastric adenocarcinoma cell lines to chemotherapy [20–23]. Therefore, XIAP is considered as a potential target for gastric adenocarcinoma therapy [24]. Notably, recent reports and our previous study showed that primary liver cancer, which usually produces AFP, also expresses high level of XIAP. The expression levels of XIAP and XIAP-associated factor-1(XIF1) also positively correlated with survival in primary liver cancer patients [25, 26]. Another study indicated that colon cancers express high levels of XIAP were prone to metastasize to liver [27]. The findings from Amemiya et al suggested that the c-Met/HGF system plays a promoting role in the progression and metastasis of AFP-producing gastric adenocarcinoma cells [12]. Therefore, a high level expression of XIAP in gastric adenocarcinoma cells, which reduces cancer cell apoptosis, could be a reason for the poor prognosis in the AFP-producing gastric adenocarcinoma patients.

Insulin-like growth factor-I receptor β (IGF-Irβ) belongs to Insulin-like growth factor (IGF) signaling system and plays an important role in gastric adenocarcinoma [28]. It was shown that the suppression of IGF-Ir causes apoptosis and reduces proliferation of gastric adenocarcinoma cells [29, 30]. Blocking IGF-Ir down-regulates VEGF ligand expression in gastric adenocarcinoma cell line, leading to neovascularization [29]. Similarly, the AFP-producing gastric adenocarcinomas featured much more frequent VEGF-C expression and higher micro-vessel density than AFP non-producing gastric adenocarcinomas [11]. A study using human gastric adenocarcinoma xenografted mice showed the blockade of IGF-Ir could enhance the chemotherapy and radiation therapy [31]. These studies supported that high level expression of IGF-Ir promotes tumor growth and metastasis in gastric adenocarcinoma.

Based on three independent prognostic factors, we established a risk model to stratify the AFP-producing gastric adenocarcinoma patients and demonstrated 2 subgroups in AFP-producing gastric adenocarcinoma. Based on IPA and GSEA analysis, the signaling pathways and proteins altered in these two subgroups were substantially different and a distinct signaling pathway and network involved in each subgroup. These molecular differences could partly explain why some AFP gastric adenocarcinoma patients live longer [6, 32]. Those proteins and pathways could be potential clinical biomarkers and therapeutic targets in AFP-producing gastric adenocarcinoma.

In conclusion, AFP producing gastric adenocarcinoma demonstrates aggressively clinical and biological behaviors. As rapidly progress in personalized medicine, targeted treatments given to specific gastric adenocarcinoma patient groups are increasingly important. AFP producing gastric adenocarcinoma is a special subtype of gastric adenocarcinoma that should be treated separately. In the current study, potential protein markers were identified and cellular pathways involved in AFP-producing gastric adenocarcinoma were suggested. Due to the low incidence and small cohort, our results need to be further validated in a larger cohort. If confirmed, future studies of the roles of these markers are warranted.

## MATERIALS AND METHODS

### Patients’ selection and follow-up

As described in the previous study [9], 45 out of 634 gastric adenocarcinoma patients with increased serum AFP were included in AFP producing gastric adenocarcinoma group while 589 patients with normal levels were included in AFP non-producing gastric adenocarcinoma group. All these patients underwent surgical resections at The First Hospital of Jilin University (Jilin, China) between January, 2009 and December, 2012. In this study, several criteria were applied for selection of the cases: (1) both fresh frozen tissue sample and serum sample (collected during the week before surgery) available in our tissue bank; (2) completely clinical data and follow-up information; and (3) patients deceased due to cancer recurrence or distant metastasis. Based on the criteria, total 77 cases, including thirty-two AFP producing gastric adenocarcinoma cases and 45 stage-matched AFP non-producing gastric adenocarcinoma controls were selected for this study. All cases and controls had the following clinical information available: age, gender, preoperative serum AFP level, resection method, localization, tumor size, histological type, differentiation degree, vessel invasion, lymph mode metastasis, liver metastasis and TNM stage. The TNM stage of the gastric adenocarcinoma was defined according to the American Joint Committee on Cancer (AJCC) Cancer Staging Manual, 7th Edition [33].

The patients were followed up every 3 month at the Department of Gastrointestinal Surgery, following standard follow-up procedures [34], with last follow-up being July 22, 2015. Relapse was defined as local recurrence or distant metastasis. The survival period was calculated from the date of surgery to the last day of follow-up or the date of death. The clinicopathological characteristics and follow-up data of the patients were summarized in Table 1.

### Serum and tissue sampling

The tumor tissues were frozen in liquid nitrogen within 30 min of removal followed by immediate pathological examination. Tumor samples of 3×3×5 mm^3^ were taken from areas without gross necrosis. Serum samples were collected before surgery through venipuncture. All Samples were kept at -80°C until analysis.

### Electrochemiluminance immunoassay

To confirm the AFP status of these 77 patients, serum AFP and four other tumor markers, including CA-724, CA199, CEA, and CA125, were re-measured 3 times using the electrochemiluminance immunoassay (ECLIA) method on a CobasTM immunoassay analyzer (Roche Diagnostics GmbH, D-68305, Mannheim). Serum AFP levels of equal or more than 7 ng/ml was defined as AFP producing gastric adenocarcinoma, according to manufacturer's protocol.

### Protein pathway array analysis

As previously described [16], fresh frozen samples were lysed by 1 ml of cell lysis buffer (Cell Signaling Technology, Danvers, MA) and protease/phosphatase inhibitor cocktail (Roche Applied Science, Indianapolis, IN). The tissue lysate was sonicated with 3 cycles of 15 seconds on icy water. The lysate samples were centrifuged at 14,000 rpm for 30 minutes at 4°C and supernatant from each sample was used for subsequent study. Protein concentrations were determined using BCA Protein Assay kit (PIERCE, Rockford, IL). Protein lysate of 300 μg was loaded in each well of a 10% SDS polyacrylamide gel. The proteins were separated by standard electrophoresis and transferred onto nitrocellulose membrane.

The multiplex immunoblot was performed using 286 antibodies, which are protein-specific or phosphorylation site-specific (Supplementary Table 1–2). The antibodies were divided into 9 sets, each containing 26–36 antibodies. For the first set of 36 primary antibodies, a mixture of 2 antibodies in the blocking buffer were added to each channel and then incubated at 4°C overnight. The membrane was then washed with Tris-buffered saline followed by Tris-buffered saline-Tween-20, and was further incubated with secondary antibody for 1 hour at room temperature. The membrane was developed with chemiluminescence substrate (Immun-Star HRP Peroxide Buffer/Immun-Star HRP Luminol Enhancer, Bio-Rad), and signals were detected using the ChemiDoc XRS System (Bio-Rad). The membrane was then stripped off using stripping buffer (Restore Western blot stripping buffer, Thermo Scientific, Rockford, IL) and the resulted membrane was used to detect a second set of primary antibodies, as described above. Each membrane was blotted with 3 sets of antibodies.

For PPA analysis, the signals of individual protein were determined by densitometric scanning (Quantity One software package, Bio-Rad), in which the background was locally subtracted from raw protein signal. Then the background-subtracted intensity was normalized using the “global median subtraction” method to reduce variation among different experiments. Here, the intensity of each protein was divided by the total intensities of all proteins from the same sample and then multiplied by averaged intensities of all proteins [16]. Normalized protein intensity was plotted with log scale before statistical analysis.

### Statistical analysis

The Significant Analysis of Microarray tool (https://github.com/MikeJSeo/SAM,) and a two-tailed Welch's t-test were used to identify proteins differentially expressed between AFP producing and non-producing gastric adenocarcinoma. Multi Experiment Viewer version 4.9 (http://sourceforge.net/projects/mev-tm4) was used to perform the unsupervised hierarchical clustering analysis. SPSS version 19.0 software (SPSS Inc., Chicago, IL) was used for statistical analysis. The Chi-square Test, Fisher exact test and Spearman test were used for correlation tests. Kaplan-Meier survival and log-rank analyses were used for survival analysis. In order to determine the risk factors associated with relapse-free survival (RFS) and overall survival (OS), univariate and multivariate analyses were performed using Cox regression method and Wald tests to assess the significance. For all statistical analysis, a p value less than 0.05 (p<0.05) was considered significant.

### Signaling network analysis

Ingenuity Pathway Analysis (IPA, desktop Version 1.0, www.ingenuity.com) was used for signaling network analysis. Both the fold differences of protein abundance between AFP producing and AFP-nonproducing gastric adenocarcinoma and gene names of the corresponding proteins identified by PPA were imported into IPA. By searching data base, IPA used a Fisher's exact test to determine which pathways were significantly linked to the input gene set. In AFP producing gastric adenocarcinoma group, the analysis aim to correlate signaling pathways and the clinical factors.

Gene-Set Enrichment Analysis (GSEA, http://www.broadinstitute.org/gsea/index.jsp) was used for network exploratory analyses in AFP producing gastric adenocarcinoma group. The advantage of the GSEA is that all proteins were ranked (not just the statistically significant) and significance was determined using a running-sum statistic for the whole gene sets [35]. We followed the previous study procedure [36]. Gene sets were taken from gene sets in the C2 and C5 sections of the Molecular Signatures Database (MSigDB). Analyses were run with 1000 permutations of gene sets (size 15–500) by using the signal to noise ranking metric. The names of the gene sets in MSigDB are based on the original data sources [37]. The IPA was employed to curate the enriched MSigDB gene sets to accurately reflect their specific biological processes.

## SUPPLEMENTARY FIGURES AND TABLES




