# Supplementary material for: Protein profiling of alpha-fetoprotein producing gastric adenocarcinoma
Source: Oncotarget. 2016 Apr 4;7(19):28448–59. doi: 10.18632/oncotarget.8571 (PMC5053738; doi:10.18632/oncotarget.8571)
Supplement: Supplementary file 1 [file oncotarget-07-28448-s001.pdf]

SUPPLEMENTARY FIGURE AND TABLES

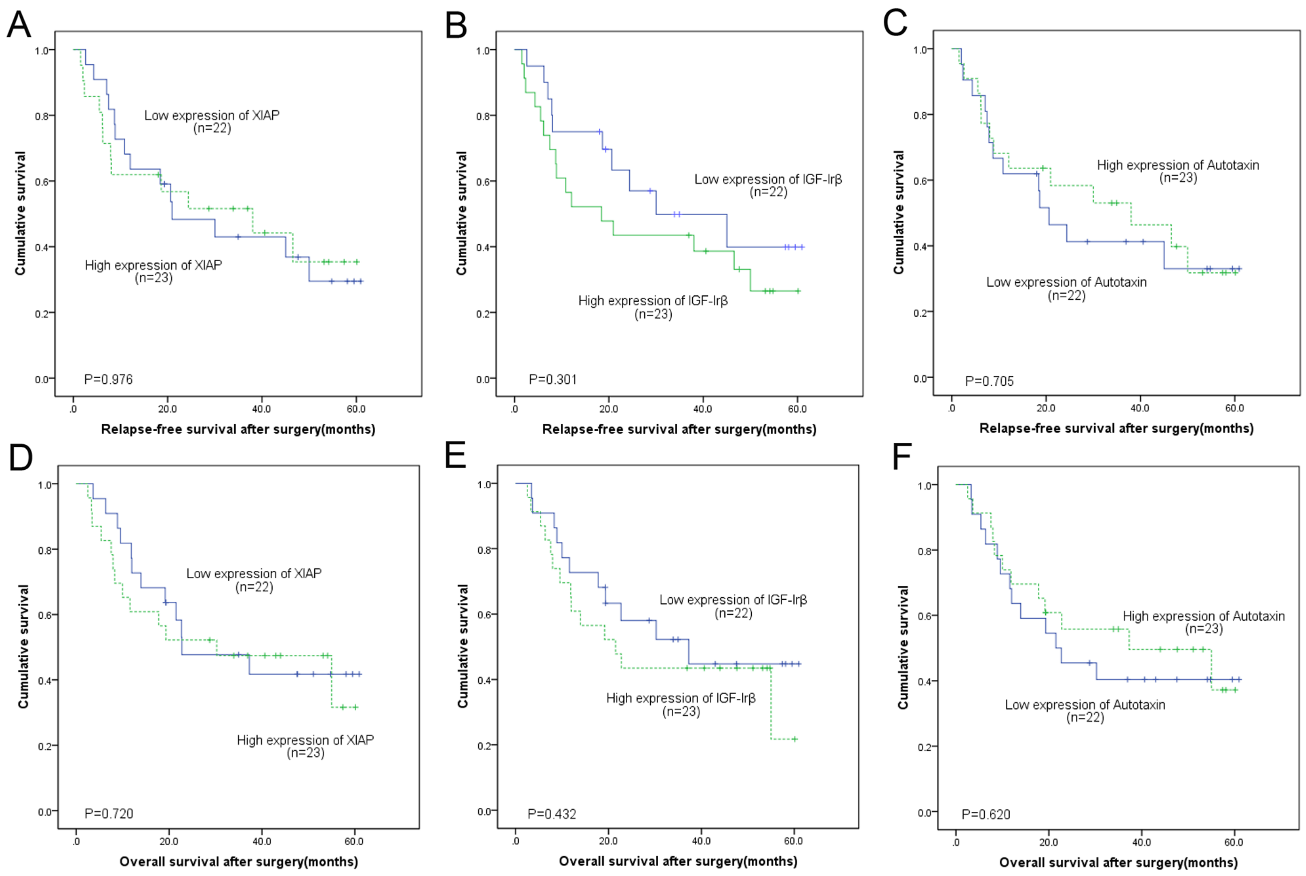

Supplementary Figure S1: In AFP non-producing group, the Kaplan–Meier and log-rank survival analysis of the relapse-free survival A-C. and overall survival D-F. showed that the group with high level expression of XIAP (A, D), IGF-Irβ (B, E) and Autotaxin(C, F) was not associated with poor prognosis.

Supplementary Table 1: Univariate Cox analyses of predictive factores for relapse-free survival in 77 gastric cancer patients

Variables	B	p value	HR	95.0% CI	
				Lower	Upper
**Sex**	0.589	0.066	1.802	0.962	3.372
**Age at operation**	-0.006	0.617	0.994	0.971	1.018
**Drink**	-0.030	0.926	0.971	0.520	1.812
**Smoke**	-0.194	0.509	0.824	0.464	1.464
**Family history of cancer**	-0.484	0.171	0.616	0.308	1.233
**Tumor location**	0.073	0.681	1.075	0.761	1.519
**Gastrectomy**	-0.080	0.787	0.923	0.516	1.652
**Histological grade**	-0.154	0.638	0.857	0.452	1.627
**Tumor size**	-0.041	0.502	0.960	0.851	1.082
**Lymph node metastasis**	1.013	0.033*	2.754	1.087	6.974
**TNM stage**	0.441	0.004*	1.553	1.153	2.093
**Vascular invasion**	-0.461	0.330	0.631	0.250	1.593
**AFP positive**	0.622	0.033*	1.863	1.053	3.297

Statistically significant as an independent predictor of survival (*p<0.05)
B, regression coefficients; HR, hazard ratio; CI, confidence interval

Supplementary Table 2: Univariate Cox analyses of predictive factores for overall survival in 77 gastric cancer patients

Variables	B	p value	HR	95.0% CI	
				Lower	Upper
**Sex**	0.345	0.284	1.412	0.751	2.656
**Age at operation**	-0.005	0.664	0.995	0.972	1.018
**Drink**	0.014	0.964	1.014	0.548	1.877
**Smoke**	-0.152	0.593	0.859	0.491	1.502
**Family history of cancer**	-0.381	0.207	0.683	0.377	1.235
**Tumor location**	0.154	0.595	1.166	0.661	2.058
**Gastrectomy**	-0.097	0.731	0.908	0.523	1.576
**Histological grade**	-0.198	0.539	0.820	0.436	1.543
**Tumor size**	-0.028	0.630	0.972	0.866	1.091
**Lymph node metastasis**	0.995	0.035*	2.704	1.071	6.825
**Curative sugury**	1.672	0.000*	5.323	2.282	12.418
**TNM stage**	0.691	0.000*	1.996	1.409	2.827
**Vascular invasion**	-0.476	0.313	0.621	0.247	1.565
**AFP positive**	-0.695	0.014*	0.499	0.287	0.866

Statistically significant as an independent predictor of survival (*p<0.05)
B, regression coefficients; HR, hazard ratio; CI, confidence interval

Supplementary Table 3: Proteins differentially expressed between AFP positive and non-positive gastric cancer tissues

	Protein	GENE ID	Fold change	q-value	p-value
**Up-regulated***	cyclin D1(M-20)	**CCND1**	4.422	0.000	**0.000**
	RANKL(FL-317)	**TNFSF11**	2.900	0.000	**0.000**
	LSD1 (1B2E5)	**AOF2**	2.678	0.000	**0.000**
	Autotaxin(E-12)	**ENPP2**	2.504	0.000	**0.002**
	Calpain 2	**CAPN2**	2.471	0.000	**0.000**
	stat 3(C-20)	**STAT3**	2.549	0.000	**0.002**
	XIAP(48/hILP)	**XIAP**	2.313	0.000	**0.002**
	IGF-Irβ	**IGF1R**	1.939	0.000	**0.000**
	Bcl-2(C-2)	**BCL2**	1.837	0.000	**0.001**
**Down-regulated***	BID (C-20)	**BID**	1.936	0.000	**0.003**
	ASC(N-15)-R	**ASC**	2.778	0.000	**0.000**

*The expression of the above listed proteins were either significantly up or down-regulated in AFP positive gastric caner patients tissue
Statistically significant in unpaired t test ($p<0.05$) and SAM analysis ($q<5\%$).

Supplementary Table 4: Univariate Cox analyses of predictive factores for relapse-free survival in AFP positive 32 gastric cancer patients

Variables	B	p value	HR	95.0% CI	
				Lower	Upper
Sex	0.984	0.058	2.676	0.967	7.405
Age at operation	-0.296	0.491	0.744	0.320	1.728
Drink	-0.002	0.996	0.998	0.388	2.565
Smoke	-0.120	0.778	0.887	0.383	2.050
Family history of cancer	-0.307	0.468	0.735	0.321	1.687
Tumor location	-0.025	0.919	0.976	0.608	1.566
Gastrectomy	-0.162	0.708	0.850	0.365	1.982
Histological grade	-1.133	0.047*	0.322	0.106	0.984
Tumor size	-0.320	0.463	0.726	0.309	1.707
Lymph node metastasis	0.254	0.099	1.289	0.953	1.744
TNM stage	0.329	0.046*	1.390	1.097	2.000
Vascular invasion	0.046	0.941	1.048	0.309	3.550
cyclin D1(M-20)	0.300	0.485	1.350	0.582	3.132
RANKL(FL-317)	-0.455	0.290	0.634	0.273	1.474
LSD1 (1B2E5)	-0.129	0.764	0.879	0.379	2.039
Autotaxin(E-12)	0.933	0.041*	2.543	1.039	6.226
Calpain 2	0.256	0.551	1.291	0.558	2.988
stat 3(C-20)	0.004	0.992	1.004	0.432	2.335
XIAP(48/hILP)	1.023	0.022*	2.782	1.162	6.661
IGF-Irβ	0.992	0.031*	2.697	1.096	6.639
Bcl-2(C-2)	0.179	0.678	1.196	0.513	2.788
BID (C-20)	0.162	0.706	1.176	0.506	2.733
ASC(N-15)-R	-0.303	0.485	0.739	0.316	1.728

Statistically significant as an independent predictor of survival (*$p<0.05$)
B, regression coefficients; HR, hazard ratio; CI, confidence interval

Supplementary Table 5: Univariate Cox analyses of predictive factores for Overall survival in AFP positive 32 gastric cancer patients

Variables	B	p value	HR	95.0% CI	
				Lower	Upper
Sex	0.533	0.263	1.703	0.670	4.328
Age at operation	0.002	0.886	1.002	0.972	1.033
Drink	-0.096	0.828	0.908	0.381	2.167
Smoke	0.072	0.855	1.075	0.497	2.327
Family history of cancer	-0.098	0.786	0.906	0.446	1.841
Tumor location	0.088	0.696	1.091	0.703	1.694
Gastrectomy	-0.091	0.803	0.913	0.447	1.866
Histological grade	-1.048	0.038*	0.351	0.130	0.943
Tumor size	-0.099	0.238	0.906	0.769	1.067
Lymph node metastasis	0.314	0.041*	1.368	1.012	1.849
TNM stage	0.457	0.014*	1.579	1.096	2.274
Vascular invasion	-0.009	0.988	0.991	0.297	3.312
cyclin D1(M-20)	0.308	0.436	1.360	0.627	2.950
RANKL(FL-317)	-0.576	0.147	0.562	0.258	1.223
LSD1 (1B2E5)	-0.137	0.555	0.872	0.554	1.374
Autotaxin(E-12)	1.215	0.007*	3.371	1.401	8.115
Calpain 2	0.396	0.316	1.486	0.685	3.223
stat 3(C-20)	-0.161	0.688	0.851	0.388	1.867
XIAP(48/hILP)	0.979	0.016*	2.661	1.200	5.901
IGF-Irβ	0.927	0.033*	2.527	1.077	5.930
Bcl-2(C-2)	0.432	0.275	1.541	0.709	3.347
BID (C-20)	0.163	0.678	1.178	0.544	2.547
ASC(N-15)-R	-0.404	0.311	0.668	0.306	1.459

Statistically significant as an independent predictor of survival (*$p<0.05$)
B, regression coefficients; HR, hazard ratio; CI, confidence interval

Supplementary Table 6: Univariate Cox analyses of predictive factores for relapse-free survival in 45 AFP non-positive gastric cancer patients

Variables	B	p value	HR	95.0% CI	
				Lower	Upper
Sex	0.481	0.262	1.617	0.699	3.744
Age at operation	-0.005	0.767	0.995	0.961	1.030
Drink	-0.072	0.867	0.931	0.403	2.150
Smoke	-0.078	0.850	0.925	0.411	2.079
Family history of cancer	-0.574	0.441	0.563	0.131	2.424
Tumor location	0.128	0.614	1.136	0.692	1.867
Gastrectomy	0.127	0.754	1.135	0.514	2.506
Histological grade	0.492	0.234	1.635	0.728	3.673
Tumor size	0.018	0.848	1.018	0.845	1.227
Lymph node metastasis	0.426	0.017*	1.531	1.079	2.172
TNM stage	0.672	0.010*	1.959	1.177	3.259
Vascular invasion	-0.947	0.199	0.388	0.092	1.644
cyclin D1(M-20)	0.029	0.941	1.030	0.473	2.239
RANKL(FL-317)	-0.443	0.263	0.642	0.296	1.394
LSD1 (1B2E5)	-0.909	0.030*	0.403	0.177	0.914
Autotaxin(E-12)	-0.149	0.706	0.862	0.399	1.863
Calpain 2	0.335	0.413	1.398	0.626	3.122
stat 3(C-20)	0.456	0.261	1.578	0.712	3.494
XIAP(48/hILP)	-0.012	0.976	0.988	0.456	2.140
IGF-Irβ	-0.416	0.304	0.660	0.298	1.459
Bcl-2(C-2)	-0.748	0.065	0.473	0.214	1.046
BID (C-20)	0.522	0.206	1.685	0.751	3.783
ASC(N-15)-R	-0.057	0.885	0.944	0.434	2.056

Statistically significant as an independent predictor of survival (*p<0.05)
B, regression coefficients; HR, hazard ratio; CI, confidence interval

Supplementary Table 7: Univariate Cox analyses of predictive factores for overall survival in 45 AFP non-positive gastric cancer patients

Variables	B	p value	HR	95.0% CI	
				Lower	Upper
Sex	0.330	0.461	1.391	0.578	3.348
Age at operation	-0.005	0.793	0.995	0.960	1.031
Drink	0.077	0.862	1.080	0.451	2.590
Smoke	-0.159	0.707	0.853	0.374	1.949
Family history of cancer	-0.749	0.227	0.473	0.140	1.595
Tumor location	0.251	0.358	1.285	0.753	2.195
Gastrectomy	0.042	0.921	1.042	0.460	2.361
Histological grade	0.440	0.305	1.553	0.669	3.602
Tumor size	0.019	0.843	1.019	0.846	1.228
Lymph node metastasis	0.515	0.010*	1.674	1.131	2.479
TNM stage	1.085	0.003*	2.959	1.453	6.024
Vascular invasion	-0.885	0.232	0.413	0.097	1.759
cyclin D1(M-20)	0.485	0.240	1.624	0.724	3.644
RANKL(FL-317)	-0.673	0.102	0.510	0.228	1.142
LSD1 (1B2E5)	-0.901	0.037*	0.406	0.174	0.945
Autotaxin(E-12)	-0.199	0.620	0.820	0.373	1.800
Calpain 2	0.233	0.567	1.263	0.568	2.807
stat 3(C-20)	0.423	0.301	1.526	0.685	3.399
XIAP(48/hILP)	0.144	0.720	1.154	0.526	2.533
IGF-Irβ	-0.316	0.434	0.729	0.330	1.610
Bcl-2(C-2)	-0.702	0.089	0.496	0.220	1.114
BID (C-20)	0.325	0.426	1.385	0.621	3.086
ASC(N-15)-R	0.014	0.973	1.014	0.457	2.251

Statistically significant as an independent predictor of survival (*p<0.05)
B, regression coefficients; HR, hazard ratio; CI, confidence interval

Supplementary Table 8: Clinicopathological characteristics of high and low risk score groups in AFP producing gastric cancer

Risk score	High risk	Low risk	p value
**cohort size**	16(50%)	16(50%)	0.7
**mean age(years)**	59±11.09	61.38±11.31	0.36
**Gender(M/F)**	13/3	13/3	0.70
**drink(Y/N)**	6/10	3/13	0.217
**Smoke(Y/N)**	8/8	7/9	0.5
**tumor size(cm)**	5.31±1.22	7.00±3.18	0.06
**nerve invasion(Y/N)**	10/6	6/10	0.144
**vascular invasion(Y/N)**	12/2	12/2	0.7
**tumor site**			
Poximal(cardia or fundus)	6	6	0.7
Distal(antrum or coupus)	10	10	
**TNM stage**			
Ib	0	1	0.54
IIb	1	3	
IIIa	1	1	
IIIb	4	1	
IIIc	7	8	
IV	3	2	
**outcome**			
**Relapse(Y/N)**	16/0	11/5	0.022*
**status at last folling up**			
Deceased	16	10	
Living	0	6	0.009*

*p<0.05

Supplementary Table 9: Antibody targets specific for phosphorylation

Antibody targets specific for phosphorylation				
p-AKT (Ser473)	p-ERK5 (Thr218/Tyr220)	**p-P44/42 MAPK (Erk1/2)(Thr202/ Tyr204)**	**p-PKCa/b II (Thr638/641)**	p-STAT 3 (Ser727)
p-b-Catenin (Ser33/37/Thr41)	p-FAK (Tyr397)	**p-P53 (Ser392)**	**p-PKCδ (Thr505)**	p-STAT5 (Tyr694)
p-CDC2 (Tyr15)	**p-GSK-3a/b (Ser21/9)**	**p-P70 S6 kinase (Thr389)**	p-PTEN (Ser380)	
p-c-Jun (Ser73)	p-JNK(G-7)	p-P90RSK (Ser380)	**p-Rb (Ser780)**	
p-CREB (Ser133)	p-Met (Tyr1234)	**p-PDK1 (Ser241)**	p-Rb (Ser807/811)	
p-eIF4B (Ser422)	p-P38 MAPK (Thr180/ Tyr182)	**p-PKCa (Ser657)**	p-Smad1/5 (Ser463/465)	

Antibodies with detectable expression in samples of two groups are in bold. β-actin and glyceraldehyde-3- phosphate dehydrogenase(GAPDH) served as internal control.
*Differentially expressed proteins between AFP producing and non-producing gastric cancer patients.

Supplementary Table 10: Antibody targets specific for non-phosphorylation

See Supplementary File 1
