# Supplementary material for: Protein profiling of alpha-fetoprotein producing gastric adenocarcinoma
Source: Oncotarget. 2016 Apr 4;7(19):28448–59. doi: 10.18632/oncotarget.8571 (PMC5053738; doi:10.18632/oncotarget.8571)
Supplement: Supplementary file 2 [file oncotarget-07-28448-s002.docx]

Table10  **Antibody targets speciﬁc for non-phosphorylation**

| **14-3-3 beta** | **Cdc2 p34** | **Dnmt1** | **Glutamine synthetase** | **ITF** | NF-kB p52 | PSCA | TGF-b |
| --- | --- | --- | --- | --- | --- | --- | --- |
| **ADAM10** | **Cdc25B** | **DPYD** | **GSTP1** | **Jagged1** | NF-kB p65 | **PSM** | TIMP-3 |
| ADAM8 | Cdc25C | DRG1 | HCAM | JAK2 | NHERF-2 | PSTPIP1 | TIP30 |
| **ADH** | Cdc42 | E2A | **HDAC1** | JNK1 | Nkx-3.1 | **PTEN** | TIRAP |
| AIM2 | **Cdk2** | E2F1 | **HER2/ErbB2** | KAI 1 | **NM23-H1/2/3** | Rab 7 | TNFa |
| **Akt** | **Cdk4** | **E-cadherin** | HES1 | **Keratin 10** | NMT1 | Raf-B | TNF-R2 |
| ALG-2 | **Cdk6** | **Eg5** | HGF | KiSS-1 | **NOS2** | RAGE | tPA |
| **Annexin A1** | Cdx2 | **EGFR** | HIF-1a | KLF6 | **Notch4** | **RANKL*** | TRAF6 |
| ASCL1 | **c-Fms/CSF-1R** | **eIF4B** | HIF-2a | K-Ras | **NQO1** | Rap1 | TS |
| **ASC-R*** | Chk1 | **Endoglin** | **HIF-3a** | LKB1 | **ODC** | **Reg IV** | **tsg101** |
| ATF-1 | **c-IAP2** | ENT1 | Hint | **LSD1*** | OPN | RHAMM | TTF-1 |
| a-Tubulin | CKR-7 | Ep-CAM | HMG-1 | **L-selectin** | p14 | **RhoA** | Twist |
| Aurora A/AIK | Clusterin | EphB2 | HNF-3a | Lyn | p16 | Ribosomal protein L6 | Tyro3 |
| **Autotaxin*** | COL1A2 | Epo | HoxC11 | **Maspin** | **p27** | RIP | uPA |
| **Axin** | Connexin 43 | ERa | H-Ras | **MAT IIb** | **P2X7** | RUNX3 | uPAR |
| Bad | **Cox-2** | ERb | HSL | MDM2 | **p38β** | **SK3** | VAP-1 |
| **Bak** | **cPKCa** | **ERCC1** | HSP 27 | **Mesothelin** | **p44/42 MAPK**  **(Erk1/2)** | SLUG | V-ATPase H |
| Bax | CREB | E-selectin | **HSP70** | **MetAP-2** | P504S | Smad4 | VCAM-1 |
| **Bcl-2*** | cSHMT | **FactorXIII B** | **Hsp90** | **MetRS** | p53 | Smad7 | VEGF |
| Bcl-6 | **CTGF** | **FAH** | **ICAM-1** | **MGr1-Ag** | p63 | **Snail** | **Vimentin** |
| **Bcl-xL** | **CTLA-4** | **FAS** | IDO | MMP-13 | p73 | **SOD-1** | VSV-G |
| **BECN1** | **CUL-1** | **FEN-1** | IFN-g | **MMP-2** | Pannexin-1 | SPAK | Wnt-1 |
| **BID*** | **CX3CR1** | FGF-8 | IGFBP5 | MMP-7 | Patched | SRC-1 | **WT1** |
| BMP-2 | **Cyclin B1** | FGFR-4 | **IGF-Irβ*** | MMP-9 | Pax-2 | **Stat1** | **XIAP*** |
| **Calpain2*** | **Cyclin D1*** | FKHR | IL-11 | MSR | PC2 | **Stat3*** | YB-1 |
| **Calpastatin** | Cyclin E | FLIPS/L | **IL-18** | **MTA1** | P-cadherin | SUGT1 | β3 Tubulin |
| **Calretinin** | **Cytokeratin18** | **Flt-3/Flk-2** | IL-1b | MTHFD1 | **PCNA** | Survivin | β-Catenin |
| CaMKKa | Cytokeratin 19 | **FOXM1** | **IL-3Ra** | MTHFD2 | **PDEF** | Syk |  |
| CARD12 | Cytokeratin 5 | FTa | **IL-6** | MTHFR | PEDF | Tak1 |  |
| **Caspase-1** | DACH1 | FUS/TLS | IL-8 | NALP1 | PERK | Tau |  |
| **Cathepsin B** | **DARPP-32** | Fusin | **IL-8RA** | N-cadherin | PKCε | TCF-1 |  |
| CD10 | DDB2 | **Galectin-3** | **Integrin a4** | NFATc1 | Plk | **TDP1** |  |
| CD33 | DHFR | GLP-1R | **IRF-1** | **NF-kB p50** | PRL-3 | **TFIIH p89** |  |

Antibodies with detectable expression in samples of two groups are in bold. β-actin and glyceraldehyde-3- phosphate dehydrogenase(GAPDH) served as internal control.

*Differentially expressed proteins between AFP producing and non-producing gastric cancer patients.
